# Correlation of Condylar Translation During Maximal Mouth Opening with Presence of Signs of Temporomandibular Joint Disorders in an Asymptomatic Population of 18-25 Years Age Group of Northern India

**DOI:** 10.2174/1745017901814010770

**Published:** 2018-09-28

**Authors:** Deepak Gupta, Soheyl Sheikh, Shambulingappa Pallagatti, Ravinder Singh, Amit Aggarwal

**Affiliations:** Department of Oral Medicine and Radiology, M.M. College of Dental Sciences and Research, Mullana, Ambala, Haryana, India

**Keywords:** Temporomandibular joint, Temporomandibular joint disorders, Anterior translation, Subluxation, Inter-incisal distance, Maximal mouth opening

## Abstract

**Objective(s):**

The objective of this study was to determine the frequency of “subluxation” and presence of clinical signs of Temporomandibular Joint Disorder (TMD) in asymptomatic individuals and its distribution according to age and sex.

**Materials and Methods:**

The material investigated comprised of 200 asymptomatic subjects with 400 joints. The subjects were divided into two groups of 18-25 years and 50-60 years of age consisting of equal number of males and females. Clinical examination involved measurement of maximal inter-incisal distance, joint sounds and deviation. For radiological examination, Temporomandibular Joint (TMJ) open mouth close mouth view option (TMJ1/2) was used on a Digital Panoramic Machine. All the radiographs were traced to assess subluxation and anterior translation of the condyle. The statistical analysis was carried out using Statistical Package for Social Sciences (*SPSS* Inc., Chicago, IL, version 15.0 for Windows).

**Results:**

The prevalence of the signs of TMDs in the asymptomatic population was found to be very high and more predominant in females as compared to males. Furthermore, the older age group had comparatively less signs of TMDs. It was of interest that the subjects presenting with clinical signs of TMD were significantly less as compared to the subjects presenting with subluxation. The value of anterior translation was found to be more in females in the younger age group as compared to the males. Similarly, it was more in males as compared to females in older age group. But the mean anterior translation difference in females in 18-25 years and 50-60 years showed a statistically significant difference with *P*-value 0.017.

**Conclusion:**

Subluxation is a very common feature found in almost all the subjects in this study with a high prevalence. Hence, we may assume that the increased incidence of TMDs could be a direct result of the phenomena of subluxation. The decrease in mandibular length could be the cause of decreased mouth opening and increased subluxation.

## 1 INTRODUCTION

Temporomandibular Joint (TMJ) in spite of being a miniscule anatomic structure is incredibly complex.It is one of the most frequently used joints in the entire body with complex movements [1-3]. It is technically classified as a ginglymoarthrodial joint. It has a combination of hinge and sliding motions [4, 5] which makes this joint among the most complicated in the body.

“Temporomandibular Joint Disorders” (TMD) is an umbrella term comprising of disorders of the TMJ or its associated structures [6-25]. Signs and symptoms of Temporomandibular Joint Disorders (TMD) vary in their presentation and can range from simple to quite vague ones [3]. Specific signs of TMJ disorders may include clicking, popping, grating sounds in the joint or limited ability to open the mouth [9, 10]. The disorder and resultant dysfunction can also result in significant pain in the TMJ or in the preauricular region. The vague symptoms may range from earaches and headaches to even neck aches and backaches [20-25].

It is important to note that several epidemiological studies have indicated that a significantly large portion of the general population exhibit signs and symptoms of Temporomandibular Joint Disorders [2, 16, 17] with females being twice as likely as men to be affected by TMDs.

Debate on disorders of this joint are ages old, having plagued humankind throughout history with the treatment of this condition being reported even during the time of the ancient Egyptians [26].Converging information from all these debates of several disciplines has contributed to such a foundation that we speak of TMJ disorders in terms of orthopedic principles, neurophysiology of pain, malocclusion, pathophysiology of joints and muscles, and behavioral aspects of chronic pain. Unfortunately, these foundations are not yet universally endorsed or even accepted by all the members of the dental profession. In this regards, dentistry remains a somewhat fragmented profession, with each discipline having its own viewpoint about many TMD issues.

Different authors and researchers have placed varying degrees of emphasis on different hypotheses as well as different etiological factors [19]. This difference amongst specialists has led many TMD patients being subjected to different treatment modalities. They may be advised complex and invasive therapies like eminectomy, disk replacement and steroid injections in the joint to simpler, conservative management methods such as physiotherapy, counseling *etc*. Thus the patients consult one specialist after another, and in the end, the patient ends up with not really satisfactory treatment.

Working of TMJ involve both a rotational and gliding movements [2]. It is generally considered that at maximal mouth opening, the condyle does not proceed beyond the articular eminence. But of significance is the fact that the condyle does proceed beyond the apex of the articular eminence during the extreme wide opening of the mouth. This was termed as “habitual luxation” [4] and it was considered as a pathological phenomenon in which the same typical symptoms as in a genuine luxation can be detected, the difference being that habitual subluxation is considerably weaker than luxation and occurs frequently from time to time [4]. This eventually causes the strength of the locking to weaken and the patient learns on his own to replace the condyle back in the fossa without any hindrance whatsoever to the closing movement [4]. Interestingly, according to Ricketts and Wassmund, the above mentioned phenomenon can be physiological as in such cases, the condyle returns back to its normal position when the patient closes the mouth. Wassmund refers it as a “non-fixed physiological subluxation” [4]. Some of the authors have used the terms dislocation and subluxation interchangeably [8]. According to the American Academy of Orofacial pain, dislocation includes both when an individual is able on his own or needs someone else to reduce the condyle [14]. In contrast, some of the authors restrict the self-reducing condition to the term “subluxation” [15]. Nevakari in the late 1960s suggested a term “elapsiopraearticularis” [4] for the same condition. According to him, it refers to slipping of the condyle up in front of the actual joint surface of the fossa and its return to the fossa without the appearance of any signs. He also referred to this event as a pathological condition or occurrence [4].

According to a study by Boman in 1947 comprising of 900 hospital patients, 153 cases showed various kinds of joint symptoms, such as cracking, creaking and pain while there was only one case of luxation. Bauerle in 1951 found habitual luxation in 40% of the cases in his study.Wassmund in 1952 and Rantanen in 1954 expressed the opinion that “physiological subluxation” occurs in only a small number of people [4]. Nevakari *et al*. in 1960 suggested that the deficiencies in research techniques or differences in approach could have led to divergent results such as mentioned above, even though no corresponding qualitative differences existed in the materials [4].

Of significance is the fact that a large number of populations exist with signs of TMDs, but no symptoms [3, 7, 19-21]. So the question arises as to whether TMDs should be considered a physiological variation and be left alone or should treatment be induced considering them as a pathological condition, especially when the patient is asymptomatic. To the best of our knowledge, no study has been done to correlate the position of the condyle during maximal mouth opening with the presence of signs of TMDs in an asymptomatic age group. To strengthen the assumption that the luxation of the condyle may be a part of an evolutionary process, a comparative group of asymptomatic subjects in the age group of 50-60 years has also been subjected to similar investigations and the results have been compared. With this in mind, the aim of this study was to correlate the Condylar Translation during Maximal Mouth Opening with Presence of Signs of Temporomandibular Joint Disorders in an Asymptomatic Population of 18-25 years age group of Northern India.

### 1.1 Aim

The aim of this study was to correlate the Condylar Translation during Maximal Mouth Opening with Presence of Signs of Temporomandibular Joint Disorders in an Asymptomatic Population of 18-25 years age group of Northern India.

### 1.2 Objectives

1. To find the incidence of signs of TMDs in asymptomatic subjects of the age group of 18-25 years from a student population of different colleges of M.M. University, Mullana, Ambala.
2. To establish the maximal mouth opening in these subjects without any symptoms of TMDs.
3. To assess the anterior translation of condyle from summit of eminence in the radiograph during maximal mouth opening.
4. To assess the incidence of sub-luxation of condyle during maximal mouth opening in asymptomatic subjects.
5. To correlate sub-luxation of TMJ with signs of TMDs in subjects without any symptoms of TMDs.
6. To compare the findings with the findings in an asymptomatic comparative age group of 50-60 years.

## 2 MATERIALS AND METHODS

The study material comprised of 200 subjects out of whom 150 subjects were in the age range between 18-25 years with 75 males and 75 females. They were selected from the patients coming to M.M. College of Dental Sciences and Research, Mullana. None of them had any symptoms of Temporomandibular joint disorders. A separate group of 50 subjects in age range of 50-60 years with 25 males and 25 females was also evaluated. This group also did not have any symptoms of TMDs.

Subjects giving a history of any known trauma involving TMJ, having luxation or any pain in or around the region of TMJ were excluded from the study. None of the subjects had any parafunctional habits or symptoms of any dysfunction of the masticatory system.

Subjects whoever had experienced any extra-articular problem that could cause reduction of mouth opening such as face and neck abscesses or infection were excluded from the study. Subjects with conditions such as Oral Submucous Fibrosis or any surgery in the TMJ region were also excluded from the study.

These inclusion criteria were also applied to the separate group of 50 subjects in the age range of 50-60 years. Apart from these inclusion criteria, another inclusion criterion was that all the subjects were completely dentulous. Ethical approval for the study was obtained from the ethical committee of M.M. University, Mullana. All the subjects were made aware of the procedure and an informed consent was taken for the same.They were subjected to clinical and radiographic examination.

### 2.1 Clinical Examination

Each subject was seated in a dental chair with his or her head leaned slightly backwards on the headrest. All of them were subjected to clinical examination which involved the recording of three putative signs for the establishment of the presence of signs of TMDs which included Limited mandibular opening, Deviation on opening and Joint sounds.

The patients were asked three times to open the mouth as wide as possible and the greatest opening was recorded. The amount of maximal mouth opening was measured with a divider positioned in between the incisal edges of the maxillary and mandibular central incisors. Since vertical overbite was not added to the value, the term Maximal Interincisal Distance (MID) was considered. Deviation on opening was visually observed by asking the individual to close on posterior teeth and then to open slowly wide. This motion was repeated several times to watch for any deviation visually. To determine joint sounds, the diaphragm of a stethoscope was placed over the skin surface over TMJ area during the opening and closing of the mouth.

### 2.2 Radiographic Investigation

**Fig. (1):**
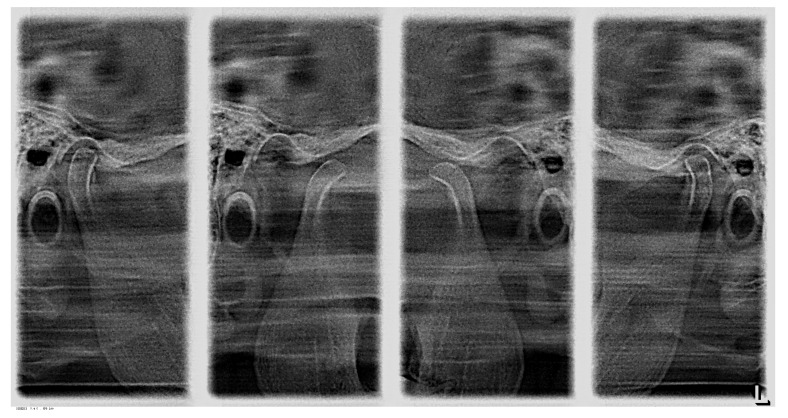
TMJ view showing bilateral subluxation.

**Fig. (2):**
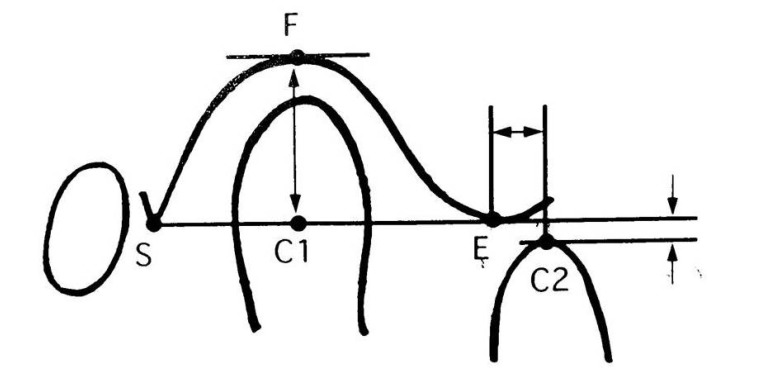
Landmarks used in the TMJ open mouth close mouth view.

The radiographic investigation was carried out with the help of Digital panoramic machine (Orthophos XG 5 DS/Ceph) manufactured by Sirona Dental Systems GmbH, Bensheim, Germany. The radiographic procedure was explained verbally to the subjects. Each individual included in the study was subjected to standard temporomandibular joint open mouth close mouth radiography. Adequate radiation protective measures were carefully followed. TMJ view was recorded (Fig. **1**) first in close mouth and then in maximum open mouth position keeping the patient in the same position. Printout of the radiographs was then obtained on a radiographic film using Kodak 5800 Dry View Laser Imager. The size of each radiograph was 11.0 inches in length and 5.7 inches in breadth (Fig. **1**). Each radiograph was then covered with tracing paper. The condyle, glenoid fossa, the articular eminence and post-glenoid spine were traced and the linear measurements were also recorded (Fig. **2**) as performed by Muto *et al*., in 1994 [20].

#### 2.2.1 The Following Landmarks were Used (Fig. **2**)

S - The point of apex of Post-glenoid spine.

E - The point of summit of articular eminence.

F - The point on the tangent to the most superior aspect of the Glenoid fossa parallel to the S-E line that intersects the fossa.

**C1**- Mid-point of the segment of the line S-E. This segment joins the two points on the condyle on the line S-E.

**C2**- The point on a line parallel to S - E line where it intersects the apex of the condyle at opening.

#### 2.2.2 The Following Linear Measurements were Used

- Distance between S and E point is the length of the fossa.
- Distance between F and C1 point is the depth of the fossa.
- Total condylar translation from the eminence is C1-C2 distance.
- Forward condylar translation from the eminence *i.e*. E-C2 distance.
- Superior or inferior shift, *i.e*. the distance between the line passing through C2 point and S-E line.

The linear values were recorded.

The statistical analysis was carried out using Statistical Package for Social Sciences (*SPSS* Inc., Chicago, IL, version 15.0 for Windows). All quantitative variables were estimated using measures of central location (mean, median) and measures of dispersion (standard deviation). Normality of data was checked by measures of skewness and Kolmogorov Smirnov tests of normality. As data was normally distributed, Unpaired t-test was applied for comparison of two groups. Qualitative or categorical variables were described as frequencies and proportions. Proportions were compared using Chi square or Fisher’s exact test whichever was applicable. To see the relationship between two variables Pearson correlation coefficient was calculated. All statistical tests were two-sided and performed at a significance level of α=.05.

## 3 RESULTS

### 3.1 Age

The mean age of males and females in the 18-25 years age group was 22.8 years and 22.9 years respectively. Similarly, the mean age of males and females in the 50-60 years age group was 55.5 years and 55.8 years respectively (Table **1**).

### 3.2 Maximal Interincisal Distance

The mean Maximal Interincisal Distance (MID) for 18-25 years of age group was 47.93±4.96 mm and 42.53±5.42 mm for males and females respectively. This difference was highly significant with *P-*value less than 0.001. Similarly, the mean MID for 50-60 years of age group was 44.54±5.67 mm and 42.0±3.93 mm in males and females respectively. This difference was not statistically significant with *P*-value of 0.071 (Table **1**). Further, the difference in mean mouth opening in between the males of the two different age groups was statistically significant with *P-* value of 0.005.

### 3.3 Reduced Mouth Opening

These were the subjects with maximal interincisal distance under 37 mm. 2.67% of males and 10.67% of females exhibited reduced maximal interincisal distance or reduced mouth opening in the younger age group of 18-25 years (Table **1**). Similarly, 8% of males and 8% of females presented with reduced mouth opening in the older age group. Reduced mouth opening in 150 subjects of 15-25 years of age group was 6.67% comprising of 10 subjects out of 150. Total number of cases of reduced mouth opening in 50-60 years of age group was 8% comprising of 4 subjects out of 50.

The difference of reduced MID in between the age groups was statistically significant for males with *P-*value of 0.049. It was not significant for females with *P*-value of 1.0

### 3.4 Joint Sounds

The total subjects presenting joint sound during auscultation with stethoscope were 119 out of 200 thus giving the prevalence of joint sounds as 59.5%.

In 18-25 years of age group, joint sounds were present in 96 out of 150 subjects, out of whom 52 were females and 44 were males (Table **1**). Thus, the joint sounds in 18-25 years age group were 64%. The number of females presenting joint sounds was more at 69.3% as compared to that of males at 58.7%. This difference was not statistically significant with *P* value of 0.174. Similarly, joint sounds were present in 23 out of 50 subjects in older age group of 50-60 years, thus giving the prevalence of 46% (Table **1**).

Further, the joint sounds present only on left side TMJ in 18-25 years of age group was 34.7% in males and 52% in females with a statistically significant difference with *P* value 0.032 (Table **2**). Similarly, in 50-60 years of age group, the joint sounds present only on left side comprised of 28% of men and 40% of women (Table **2**) with no statistically significant difference.

The joint sounds present only on right side TMJ in 18-25 years of age group was 34.7% in males and 37.3% in females. There was no statistically significant difference. Similarly, in the older age group, on the right side, the joint sounds were found in 20% of males and 28% of females respectively with no statistically significant difference (Table **2**).

### 3.5 Deviation on Opening

The deviation in 18-25 years of age group was 25.3% and 26.7% in males and females respectively with no statistically significant difference. Similarly, in 50-60 years of age group, 16% of males and 24% of females showed deviation with no statistically significant difference (Table **1**).

### 3.6 Signs of Temporomandibular Joint Disorders

The number of subjects presenting with at least one sign of TMD on the basis of Research Diagnostic criteria were 74.67% in females and 66.67% in males of 18-25 years of age group. Similarly, it was 68% in females and 52% in males in 50-60 years of age group (Table **1**).

The difference in the number of TMD cases between males and females in both the age groups was not statistically significant with *P*-value 0.2814 and 0.2488 respectively.

#### 3.6.1 Subluxation

In 18-25 years of age group, 71 males and 71 females presented with subluxation unilaterally or bilaterally. The prevalence was 94.7% in males and 94.7% in females. Similarly in older age group of 50-60 years, equal number of subjects presented with subluxation, comprising of 21 out of 25 males and 21 out of 25 females (Table **1**). The prevalence of subluxation in this age group was 84% in males and 84% in females.

#### 3.6.2 Subluxation with TMDs

In 18-25 years of age group, 63% males and 71% females had subluxation with clinical signs of TMD with no statistically significant difference. Similarly, in 50-60 years of age group, 48% of males and 56% of females had subluxation with clinical signs of TMD with no statistically significant difference.

#### 3.6.3 Subluxation without any Clinical Sign of TMDs

In 18-25 years of age group, 32% males and 24% females had subluxation but no clinical signs of TMD. Similarly, in 50-60 years of age group, 36% of males and 28% of females had subluxation but no clinical signs of TMD (Table **3**).

This difference in the prevalence of subluxation without any clinical sign of TMD between males and females in 18-25 years and 50-60 years of age group was not statistically significant with *P*-value of 0.2750 and 0.5430 respectively.

#### 3.6.4 TMD without Subluxation

In 18-25 years of age group, 4% males and 4% females had TMD signs clinically but no subluxation. Similarly, in 50-60 years of age group, 4% males and 12% females had TMD signs clinically but no subluxation (Table **3**).

The difference in the prevalence of any clinical sign of TMD but without subluxation between males and females in 18-25 years and 50-60 years of age group was not statistically significant with *P*-value of 1.00 and 0.609 respectively.

### 3.7 Anterior Translation

The distance between the condyle and the summit of the eminence *i.e*. E-C2 distance at maximal mouth opening in 18-25 years of age group was 6.52±3.66 mm in males and 6.63±3.32 mm in females (Table **4**). This difference was not statistically significant.

Similarly, the distance between the condyle and the summit of the eminence at maximal mouth opening was 5.08±4.27 mm in males and 4.75±3.49 mm in females in 50-60 years of age group (Table **4**). The difference was not statistically signigicant. But the mean anterior translation difference in females in 18-25 years and 50-60 years showed statistically significant difference with *P*-value 0.017.

## 4 DISCUSSION

Temporomandibular disorder is a collective term used for a number of clinical problems that involve the masticatory muscles, temporomandibular joint and/or associated structures [27-31]. Various authors have proposed different criteria for the diagnosis, treatment planning and for the research purpose for defining subject groups. These criteria include Research Diagnostic criteria, American Academy of Craniomandibular disorder guidelines and many more. But in this study, Craniomandibular Index criteria (CMI) described by Friction and Schiffman in 1986 was followed according to which limitation in mandibular movements, jaw deviation while opening and joint sounds were categorized under craniomandibular disorders [28, 32].

The subjects chosen in this study were in the age range of 18-25 years and 50-60 years. This subject age was selected thus because the growth period of the condyle does not affect the results of this study. The articular tubercle is not well defined until the age of 6-7 years and it assumes its adult shape at about 12 years [33]. Furthermore, the growth in height of the tubercle appears to continue throughout adolescence [4, 11, 34].

The mean age of males and females in the 18-25 years age group was 22.8 years and 22.9 years respectively. Similarly, the mean age of males and females in the 50-60 years age group was 55.5 years and 55.8 years respectively.

In the present study, the mean values for MID were more in males as compared to females in both the age groups (Table **1**). This difference in MID was highly statistically significant (*P*< 0.001) for the younger age group while it was not significant for the older age group (*P* =0.071). This gender difference was thought to be due to the fact that the maximal mouth opening, to some extent, depends on the size of the mandible [19] which is significantly greater in males as compared to females [20]. As far as the older age group was concerned, the mean maximal interincisal distance found in this study was less as compared to the younger age group. This was in agreement with the fact that after adulthood, maximal mouth opening decreases with age [19, 35]. This can be attributed to reduced laxity of the ligaments [35]. These results were in agreement with previous studies done by Gross *et al.,* and others [19, 24, 26]. But their values were less as compared to the values quoted by Muto *et al*., Wesling*et al*., and many more [28, 36].

In agreement with the findings of previous studies [3], females were found to be more prone to restricted mouth opening.

The percentage of cases presenting deviation of the mandible to either left or right side in the younger age group was on an average, the same in males and females. The same results were found in the older age group (Table **1**). The total number of subjects in the younger age group was comparatively more as compared to the older age group with prevalence of 26% and 20% respectively. These results were in contrast to the findings of Huber *et al*., who observed 45% and 50% deviation in males and females respectively [3]. On the contrary these results were more as compared to the observation of Meti M *et al*. in 2002 who observed only one case of deviation in a sample of 60 subjects [19].

TMJ sounds are often an indication of mechanical interferences within the joint. In this study, large number of subjects presented with joint sounds in the form of clicking during auscultation with stethoscope. These joint sounds were present in 119 subjects out of 200, thus giving a total prevalence of 59.5%. This was in agreement with Huber *et al*. who presented a value of 51% in males and 50% in females [3]. These results were higher as compared to most of the studies done in literature [19].

Furthermore, in this study, the younger age group was found to have more joint sounds as compared to the older age group. Within the age groups, the females significantly had more joint sounds when compared to males. This was in agreement to various authors who observed a significant difference in the prevalence of joint sounds between men and women [17, 19]. Further none of the authors has commented upon the prevalence of joint sounds in terms of left or right TMJ. The left side of the joint was found more frequently involved in joint sounds as compared to the right side in both the age groups. The prevalence of TMJ joint sounds on left side in younger age group was found to be significantly more in females as compared to males with *P*-value 0.032.

The fact of high prevalence of the signs of TMDs in this asymptomatic population [7, 8, 14, 16, 19-22], especially females, strengthens the fact of high prevalence of symptomatic female patients with TMD. However, the role of other factors which have been proposed to explain the predominance of females with TMJ dysfunction cannot be denied. These factors include points such as women being more likely to seek health care than men and the higher incidence of psychophysiologic disease, acute arthritis and headache being greater in females.

Aufdemorte and associates have proposed the role of sex hormones in pathogenesis of TMJ dysfunction [37]. Although, in our view, all these putative signs of TMDs being prevalent in females [37-43] along with the factors described above can provide a conclusive explanation for the predominance of female patients with TMJ dysfunction, additional research is still required.

In this study, the number of subjects presenting with at least one sign of TMD was more in females in both the age groups. This clearly indicates that females have more prevalence of TMD signs as compared to males even in asymptomatic subjects. In the older age group, though the females presented with TMD more commonly, the number was comparatively less as compared to the females of younger age group.

Various authors have commented on the use of different radiographic approaches for TMJ examination [25, 42, 43] which includes panoramic radiography [11, 30], computed tomography, MRI *etc*. Further according to Mohl [38] and Kaplan & Assael [39], the efficacy of diagnostic modalities can be determined by the validity and reliability [1]. In this study, after clinical assessment, radiographic examination was performed on the TMJ open mouth close mouth view option on a digital radiographic machine manufactured by Sirona. This view was chosen because it gives the TMJ of left as well as right side in both open and closed mouth position in a single image (Fig. **1**) thus reducing the time of the procedure. Since the head position, radiographic projection and the position of the mandible in centric occlusion were stable and standardized, there cannot be any radiographic alterations. In this way, the images obtained in this study were valid as well as reliable.

During radiographic assessment, the condyles translated anterior to the summit of articular eminence in almost all of the subjects. These subjects were designated as having subluxation. In only 16 cases out of 200, the condyle did not cross the summit of articular eminence.

Interestingly, it was observed that a significant number of subjects who presented with signs of TMDs showed subluxation during radiographic evaluation. Further cross relating, it was found that the subjects presenting with signs of TMD were significantly less as compared to the subjects presenting with subluxation. Hence, subluxation can be assumed to be a normal phenomenon. It was further supported by the fact that there were adequate number of subjects who presented with subluxation without any sign of TMD *i.e* 58 out of 200, while there were only 10 subjects out of 200 who had clinical signs of TMD without subluxation.

The maximal condylar movement, also termed as anterior translation was measured on the side of greatest movement. It was found that the distance between the condyle and the summit of the eminence at maximal mouth opening was almost the same in males and females (Table **4**) in the 18-25 years age group, with no statistically significant difference. This was not in accordance with older studies. On the other hand, in the older age group, the anterior translation was more in males than females. But again the difference was not statistically significant. Furthermore, it was very interesting to note that the mean anterior translation difference in females in the 18-25 years age group and the 50-60 years age group showed statistically significant difference with *P*-value of 0.017.

In earlier literature, it was concluded by several authors that the condylar translation was significantly correlated with mouth opening and that the mouth opening was found to be significantly more in males than that of females. This could be a reason why they found higher values of anterior condylar translation in males as compared to females. But in contrast, it is also commented that mouth opening depends upon the size of the mandible. Dijkstra *et al*., in 1999 commented that mouth opening not only reflects the mobility of the TMJs [40, 41], but also reflects the mandibular length [41]. As the length of the mandible in males is more, so they have more range of mouth opening.

Henceforth in our view, if the size, or more precisely, the length of the mandible is more, then the same range of mouth opening can be achieved with less of translation and/or less of angular displacement of mandible relative to the calvarium [41]. This fact is further supported by Dijkstra *et al*., in 1999 who concluded that mouth opening is significantly influenced by mandibular length and angle of mandibular opening [33, 41]. So we can say that since females have smaller mandibles, there is limited mouth opening as compared to males and henceforth they need more condylar translation to maintain the mouth opening.

## CONCLUSION

We found a statistically significant difference in Maximal Interincisal Distance of males in both the age groups. Maximal Interincisal Distance in males in the younger age group was more as compared to the older age group. Further there was no difference in Maximal Interincisal Distance in females of both the age groups. So according to our hypothesis, as females have smaller jaw length, so to maintain mouth opening to the same value, there was more translation. That is why we probably got significant difference in anterior translation in females with more translation in the younger age group. At the same time translation was not significantly different between males and females in both the age groups. Moreover, it was more in females than that of males in the younger age group. Henceforth, due to comparatively smaller jaw length, to maintain mouth opening, females compensated with more translation.

Now this gliding of the condyle to the summit of the articular eminence or even ventral to it during maximum mouth opening was of interest. As previously described in the introduction section, this phenomenon has been termed as “subluxation” rather inappropriately since this anatomically implies a pathological phenomenon. But in light of the results of this study, we can say that this phenomenon is quite common and present in asymptomatic subjects. Hence, it would not be wrong if it is commented that subluxation is not hazardous to the individual and could be considered as only a physiological phenomenon.

Further, it is well accepted that the mandible is growing smaller in size, with various commonly encountered features associated with this fact such as increase in the incidence of impacted teeth, missing teeth and malocclusion. In our view, subluxation may also be included as a feature related to the fact of the mandible becoming smaller in size in the coming generations. Accordingly, this could explain the increase in signs and symptoms of TMDs which could be a direct result of the phenomena of subluxation. Keeping these facts in mind, we can tentatively conclude that TMDs may need to be managed by more conservative methods such as exercises, physiological instructions, psychological counseling and so on, rather than focussing on more complex or invasive therapeutic methods.

## Figures and Tables

**Table 1:** Clinical and radiological parameters measured.

**Gender**	**Age Group**	**N**	**Mean M.I.D (in mm)**	**R.M.O**	**Joint Sounds**	**Deviation**	**Prevalence of Clinical Signs of TMDs**	**Subluxation**
**Male**	**18-25 years**	75	47.93±4.96	2 (2.67)	44 (58.7)	19 (25.3)	50 (66.67)	71 (94.7)
	**50-60 years**	25	44.54±5.67	2 (8)	10 (40)	4(16)	13 (52)	21 (84)
	**Total**	100	47.09±5.32	4 (4)	54 (54)	23 (23)	63 (63)	92 (92)
**Female**	**18-25 years**	75	42.53±5.42	8 (10.67)	52 (69.3)	20 (26.7)	56 (74.67)	71 (94.7)
	**50-60 years**	25	42.0±3.93	2 (8)	13 (52)	6 (24)	17 (68)	21 (84)
	**Total**	100	42.40±5.07	10 (10)	65 (65)	26 (26)	73 (73)	92 (92)

N is total number of subjects M.I.D. is Maximal Interincisal Distance R.M.O. is Reduced Mouth opening TMDs is Temporomandibular Joint Disorders Values in parenthesis denotes percentage.

**Table 2:** Joint sounds present only on left or only on right side.

**18-25 years**	**Joint Sounds**	**Present on Left Side**			**Present on Right Side**		
		**Male**	**Female**	**Total**	**Male**	**Female**	**Total**
	**Count**	26	39	65	26	28	54
	**% within gender**	34.7%	52.0%	43.3%	34.7%	37.3%	36.0%
	**Total Count**	75	75	150	75	75	150
**50-60 years**	**Count**	7	10	17	5	7	12
	**% within gender**	28.0%	40.0%	34.0%	20.0%	28.0%	24.0%
	**Total Count**	25	25	50	25	25	50

**Table 3:** Subjects with subluxation but without TMD; and subjects with TMD but without subluxation.

**Age Group**	**Gender**	**N**	**Subluxation but no TMD Cases**	**TMD but no Subluxation Cases**
18-25 years	Male	75	24 (32)	3 (4)
	Female	75	18 (24)	3 (4)
50-60 years	Male	25	9 (36)	1 (4)
	Female	25	**7 (28)**	**3 (12)**

N is number of subjects and values in parenthesis denotes percentage

**Table 4:** Maximum anterior translation of condyle.

**Gender**	**Age Group**	**N**	**Mean in mm**	**Std. Deviation**	**Student T-test** **(*P* value)**
**Male**	**18-25 years**	75	6.52	3.66	0.106
	**50-60 years**	25	5.08	4.27	
	**Total**	100	6.16	3.85	
**Female**	**18-25 years**	75	6.63	3.32	0.017*
	**50-60 years**	25	4.75	3.49	
	**Total**	100	6.16	3.46	

* p value statistically significant
